# How networks shape diversity for better or worse

**DOI:** 10.1098/rsos.230505

**Published:** 2024-05-22

**Authors:** Andrea Musso, Dirk Helbing

**Affiliations:** ^1^ Computational Social Science, ETH Zürich, Ramistrasse 81, Zürich 8092, Switzerland; ^2^ Complexity Science Hub, Josefstädter Strasse 39, Vienna 1080, Austria

**Keywords:** networks, diversity, cultural evolution, opinion dynamics, graph theory

## Abstract

Socio-diversity, the variety of human opinions, ideas, behaviours and styles, has profound implications for social systems. While it fuels innovation, productivity and collective intelligence, it can also complicate communication and erode trust. So what mechanisms can influence it? This paper studies how fundamental characteristics of social networks can support or hinder socio-diversity. It employs models of cultural evolution, mathematical analysis and numerical simulations. We find that pronounced inequalities in the distribution of connections obstruct socio-diversity. By contrast, the prevalence of close-knit communities, a scarcity of long-range connections, and a significant tie density tend to promote it. These results open new perspectives for understanding how to change social networks to sustain more socio-diversity and, thereby, societal innovation, collective intelligence and productivity.

## 1. Introduction

In his seminal work, *The Selfish Gene* [1], Richard Dawkins proposes a compelling perspective on culture and its evolution. He argues that, by viewing *memes*^1^—a collective term encompassing human ideas, opinions, behaviours and styles—as cultural counterparts to genes, one can use the foundational principles of biological evolution to explain the origins and development of cultural traits.

Biological evolution is driven by mutation and selection, acting on biology’s fundamental units: genes [2]. Mutation introduces new genetic variants into a population, which undergo selection through individual interactions. The nature of these interactions varies: some individuals engage with each other frequently, while others are predominantly isolated. Such patterns of selective interaction, commonly known as the interaction network structure, critically determine survival chances, thereby influencing which genetic variants are perpetuated through reproduction and which become extinct. In essence, the structure of the interaction network is instrumental in shaping the variety of genes present in the population, or, in other words, its bio-diversity.

Research suggests a complex relationship between the structure of interaction networks and bio-diversity. For example, certain inter-individual interaction networks can increase the advantage of fitter individuals, potentially reducing bio-diversity [2,3]. By contrast, the pronounced nestedness found in inter-species interaction networks seems to boost bio-diversity by reducing direct competition among species [4].

Socio-diversity, defined as the variety of memes (i.e. ideas, opinions, behaviours and styles) present in a society, is a social parallel to bio-diversity. Dawkins’ perspective on cultural evolution suggests that socio-diversity emerges from the interplay of imitation and innovation, acting upon culture’s basic units: memes [5,6]. Innovation, akin to biological mutation, creates new cultural variants (new memes). Imitation, as a counterpart of biological selection, determines which variants diffuse across society.

Just as the structure of species interaction networks influences bio-diversity, the structure of social interaction networks affects socio-diversity. There is substantial evidence that the network structure in which individuals are embedded [7] significantly influences the diffusion of various memes, such as obesity [8], smoking [9], cooperation [2,10,11] and product adoption [12]. Generally, clustered networks, characterized by numerous closed triangles (i.e. your friends are also friends), excel at spreading memes, such as complex behaviour, requiring repeated endorsement [12–14]. The dense local connections in these networks provide the repeated exposure necessary for such memes to take hold. Conversely, networks characterized by an abundance of long-range connections are more effective at disseminating simpler memes that need minimal reinforcement [15,16]. These far-reaching connections enable quick meme transmission across diverse network areas, facilitating rapid spread.

Network structure also significantly shapes meme creation. Efficient networks, characterized by short distances between nodes, appear to hinder the creation of radically novel memes by facilitating blind imitation [17,18]. By contrast, networks with larger average distances between nodes, seem to foster the generation of novelty by providing fewer imitation opportunities [17,18].

In summary, while there is substantial evidence on the influence of network structure on patterns of meme diffusion and creation, and these patterns clearly affect socio-diversity, research directly examining the relationship between network structure and socio-diversity is scarce. As a result, a systematic understanding of this relationship is still lacking, especially when compared with the comprehension in ecology and conservation biology of the similar relationship between network structure and bio-diversity.^2^ Crucial questions remain open: How can we measure a social network’s potential to foster socio-diversity? Which social networks enhance socio-diversity, and which ones diminish it?

This paper addresses these important questions by introducing a novel index linking network structure and socio-diversity: the *structural diversity index*. This index, derived from the theory of random walks on networks [19,20], quantifies the propensity of a network to support socio-diversity; its ability to protect unpopular memes from being crushed by more popular ones. With our novel index, understanding if a social network enhances or diminishes socio-diversity becomes straightforward: it suffices to compare the network’s index value against a benchmark (such as the complete network). If a network’s index value is higher than the benchmark, then the network promotes socio-diversity; otherwise, it hinders it. The index is designed for scalability, can handle large networks and is easily accessible through the Python package accompanying this paper.

Employing our novel index, we conducted an extensive exploration of the relationship between network structure and socio-diversity across a broad range of real-world and synthetic networks. We focused on some key network characteristics, such as the shape of the degree distribution, the edge density and the prevalence of long-range connections. Our selection of these characteristics is underpinned by four guiding hypotheses. First, networks dominated by highly connected individuals may see diminished socio-diversity as a result of the disproportionate influence on cultural spreading exerted by such individuals. Second, networks with prevalent long-range connections might display lower socio-diversity due to the homogenizing effects of such ties. The pervasive spread of global pop culture and its consequential effect on local cultures exemplifies the possible homogenizing impact of these long-range connections [21]. Third, networks with a high density of connections may bolster socio-diversity. Specifically, increasing the number of connections diminishes the influence of each individual connection and, thereby, lowers the chances of viral meme cascades [12]. Fourth, networks characterized by numerous close-knit communities may enhance socio-diversity. In fact, when these communities share homogeneous memes, they limit exposure to new memes and foster the persistence of those already adopted.

We initiated our investigation by examining two prominent synthetic network models: scale-free [22] and Watts–Strogatz [23] networks. Although these models might seem simplistic, they serve as useful tools to isolate the effect of different network characteristics. Specifically, scale-free networks offer a lens to study degree-heterogeneity, or in simpler terms, the unequal distribution of connections among nodes. Our analysis of these networks’ structural diversity index suggests that such a high disparity in connections can reduce socio-diversity. This aligns with the hypothesis that individuals with numerous connections may inadvertently suppress socio-diversity due to their pronounced influence on cultural transmission. Conversely, Watts–Strogatz networks provide a framework to understand the ramifications of long-range connections and close-knit communities. Our findings indicate that fewer long-range connections and more close-knit communities are positive for socio-diversity. This is compatible with the idea that cultural convergence towards a ‘global village’ is expected to erode socio-diversity.

Moving beyond these simplistic models, we broadened our investigation to include hundreds of real-world networks. Using the comprehensive real-world network database provided by graph-tool [24], we computed the structural diversity index of networks originating from various social contexts. Our findings reinforce that high inequality in the distribution of connections suppresses socio-diversity, while the scarcity of long-range connections and a prevalence of close-knit communities amplify it. Furthermore, we observed that a high density of connections also tends to improve socio-diversity.

Although the factors shaping socio-diversity have drawn interest from social scientists [25–29], its role in social science research has not reached the prominence of bio-diversity in ecology and conservation biology. Yet, socio-diversity has profound real-world implications [30]. On the positive side, socio-diversity is a catalyst for innovation [31], promotes cooperation [32,33], and can increase productivity [34,35]. Research shows that protecting novel and rare ideas from premature dismissal [17,36] and encouraging independent thought [37–40] can enhance a group’s ability to solve all kinds of problems, making it more ‘intelligent’, more productive and more innovative. Conversely, socio-diversity can pose challenges to group cohesion [41,42], impede effective communication [43], and erode trust [44,45]. Our research seeks to reduce the relative disparity in attention between bio- and socio-diversity by shedding light on the interplay between network structure and socio-diversity.

## 2. Results

### 2.1. Structural diversity index

Consider a social network represented abstractly by a connected undirected graph *G*. In this representation, each vertex stands for an individual, and edges symbolize undirected and mutual relationships, such as friendships, acquaintances or interactions.

For illustration, imagine that two individuals, Alice and Bob, decide to play the ‘random social exploration game’, a variation of Milgram’s celebrated small-world experiment [46]. In this game, Alice and Bob each randomly select a friend from their network and send them a letter. This letter carries a simple instruction: ‘Please choose a friend at random and forward this letter to them.’ Every recipient follows this directive, passing the letter onward within their network. As the letters get forwarded again and again, they randomly explore the social circles of both Alice and Bob. The game ends when the two letters *meet*, i.e. when they simultaneously end up in the mailbox of the same individual.

**Figure 1.**
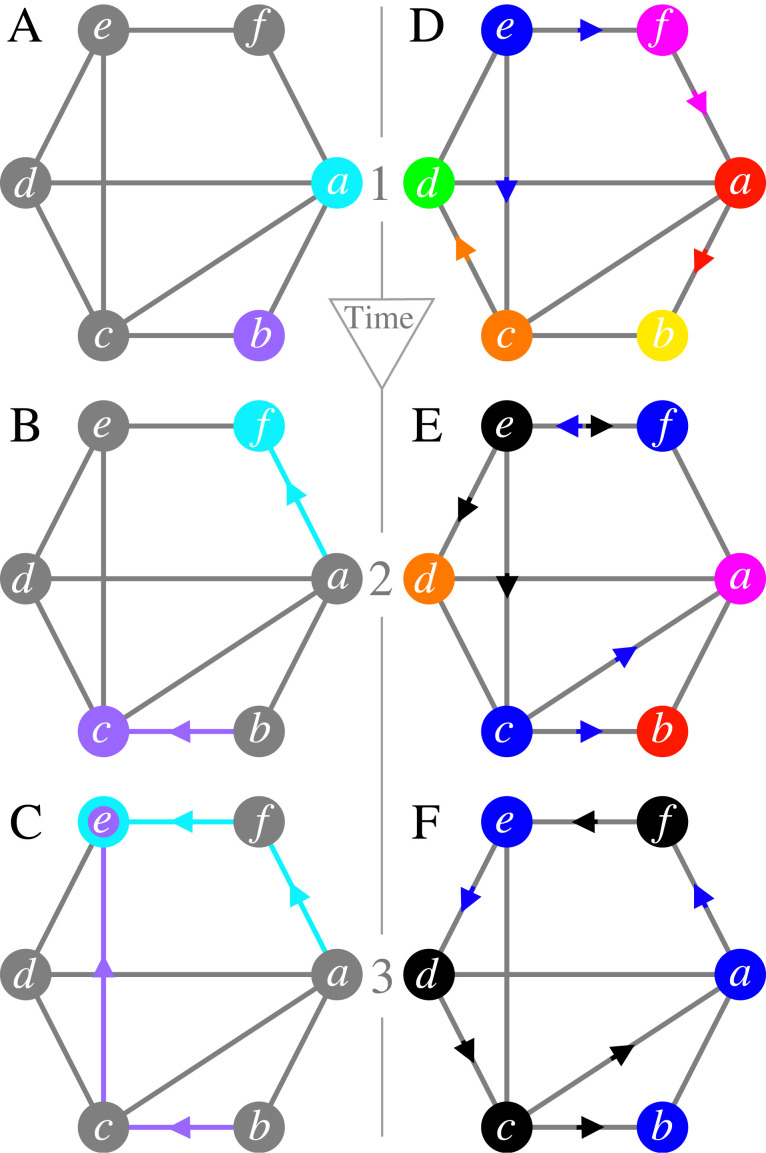
An illustration of the random social exploration game (A–C) and the progression of cultural evolution in a small social network (D–F). The network features six individuals: Alice (*a*), Bob (*b*), Carla (*c*), Darcy (*d*), Elon (*e*) and Frank (*f*). (A–C) In the random social exploration game, letters sent by Alice and Bob are randomly forwarded through the network until they meet in a mailbox (see main text for details). In this illustration, the letters’ journeys are marked by highlighted edges, converging at Elon’s mailbox. (D–F) These panels illustrate cultural evolution within our social network in three-time steps. Each panel depicts both the present meme distribution and its imminent evolution by colours. An individual’s current meme is reflected by the colour of their vertex, whereas upcoming changes are represented by the colour and direction of arrows pointing towards their vertex. For instance, Alice’s red meme in *d* transforms into Frank’s magenta meme (see *e*), as indicated by the magenta arrow pointing from Frank to Alice. It is noteworthy that in *d*, no arrows point towards Elon, suggesting he does not mimic others at *t* = 1, but instead introduces a new meme (the black meme).

As an example, consider the hexagonal-shaped social network depicted in figure 1A. This network comprises six individuals: Alice (*a*), Bob (*b*), Carla (*c*), Darcy (*d*), Elon (*e*) and Frank (*f*). In the initial phase of the random social exploration game (shown in A), Alice selects a friend at random to send her letter. Thus, her letter stands an equal chance of landing with Bob, Carla, Darcy or Frank. In this particular illustration, fate dictates the letter to be sent to Frank, as shown in B. Upon receiving the letter, Frank, too, makes a random choice, deciding to forward the letter to Elon. Simultaneously, Bob’s letter also finds its way to Elon, having first been relayed through Carla. It is at this point, in Elon’s mailbox, that the letters from Alice and Bob *meet*, marking the game’s end.

The *expected meeting time* of the network *G*, denoted 〈*M*_*G*_〉, is defined as the average number of forwards required for the letters to meet. Specifically, it is computed by conducting many repetitions of the random social exploration game, with letters starting out from different individuals and then averaging the number of forwards necessary for the letter to meet in each game iteration. Formally, the expected meeting time 〈*M*_*G*_〉 is defined as the average number of steps before two uniformly started random walks on *G* visit the same vertex simultaneously. It is a well-studied network statistic [47,48].

The structural diversity index of the network *G* is defined as the ratio between its expected meeting time 〈*M*_*G*_〉 and its number of vertices, or size, |*V*(*G*)|:

2.1 $$\Delta (G)=\frac{\left\langle M_{G}\right\rangle }{\left|V(G)\right|}.$$

On the surface, Δ(*G*) is a scale invariant measure of the ease of meeting during a random walk on the network. Although this metric is solely based on the network’s structure, it offers powerful predictions of the network’s propensity to support socio-diversity.

The fascinating relationship between the structural diversity index Δ(*G*) and socio-diversity is best understood through a simple model of cultural evolution, commonly known as the voter model [49]. As before, we have a population occupying the vertices of a network *G* with edges symbolizing various undirected and reciprocal relationships, such as friendships, acquaintances or interactions.

At the beginning, each individual *i* of the population displays a distinct meme *m*_*i*_. A meme symbolizes an individual cultural trait that can vary—such as political beliefs or musical tastes. Cultural evolution is assumed to happen at discrete time steps. During each step, individuals simultaneously modify their meme in one of two ways:

(i) by imitating the current meme of a randomly chosen neighbour, which occurs with a probability of 1 − *r*.
(ii) by inventing a novel meme that has not been previously existed, with a probability of *r*.^3^

The parameter *r*, with 0 ≤ *r* ≤ 1, is referred to as the innovation rate. It measures the equilibrium between the two key evolutionary forces of imitation and innovation. Higher values of *r* stimulate innovation, while lower values strengthen imitation.

Figure 1D–F illustrates cultural evolution in a small social network over three time-steps *t* = 1, 2, 3. In *d*, each individual displays a distinct meme, represented by the colour of its respective vertex. We focus on the journey of a specific individual, Elon. Transitioning from *t* = 1 (*d*) to *t* = 2 (*e*), we observe that Frank and Carl imitate Elon’s blue meme (indicated by blue arrows). By contrast, Elon innovates, introducing a new meme: the black meme. Globally, the meme landscape has undergone a transformation. Elon’s black meme enters the scene, the green and yellow disappear, and the blue meme gets increased attention. Advancing from *t* = 2 (*e*) to *t* = 3 (*f*), Elon opts to imitate Frank by embracing the blue meme. Concurrently, Frank, Carl and Darcy find Elon’s new meme appealing and imitate it. As a result, only two dominant memes emerge: blue and black.

In short, as cultural evolution unfolds over time, individuals either innovate (as Elon) by creating new memes or imitate (as everyone else) by adopting existing memes from their peers. These processes continually reshape the meme landscape in the population, influencing its overall socio-diversity. For instance, it is evident that the population in figure 1’s D is inherently more diverse than that in *f*. Yet, to quantify this difference in diversity, one requires a specific measurement of socio-diversity.

In this study, we adopt a well-known diversity measure in ecology: Simpson’s diversity index [50]. This index takes into account both the number of memes, as well as their relative abundance. It is defined as the probability that two randomly selected individuals in the population display different memes. The value ranges from 0 to 1. When the population’s memes are homogeneous, the probability that two randomly selected individuals exhibit different memes is small. Consequently, Simpson’s diversity index is close to 0. Conversely, when the population’s memes are diverse, the probability that two randomly selected individuals display different memes is large. Accordingly, Simpson’s diversity index is close to 1.

In the context of our cultural evolution model, Simpson’s diversity index at fixed time *t* can be computed as

2.2 $$D(t)=1-\sum_{m}p_{m}{\left(t\right)}^{2}.$$

Herein, the summation is over all memes present at time *t*, and *p*_*m*_(*t*) is the fraction of individuals who display meme *m* at that time. For illustration, the socio-diversity *D*(*t* = 1) of the (highly diverse) population portrayed in figure 1D is *D*(1) = 0.833, while that of the (more homogeneous) population in figure 1F is *D*(3) = 0.5. In summary, higher values of *D*(*t*) reflect greater socio-diversity within the population.

Returning to our fundamental research question, we can now frame it more precisely: how does the network structure *G* of a population influence its socio-diversity *D*(*t*)? Our answer is the following simple and elegant mathematical equation, which is a generalization of results by Aldous and collaborators [49,51]. This equation links the meeting time in the random social exploration game (depicted in figure 1A–C) to the long-term socio-diversity in our cultural evolution model (as shown in figure 1D–F). The derivation of this equation arguably represents the most intricate part of our analysis and can be found in Methods

2.3 $$D_{\infty }\overset{def}{=}\lim_{t\to \infty }\frac{1}{t}\sum_{s\leq t}D(s)\approx 1-e^{-2\alpha \Delta (G)}.$$

The left-hand side of the equation introduces *D*_∞_, the average population wide socio-diversity over an extended period of time (henceforth the population’s *expected socio-diversity*). This quantity is the focal point of our exploration. We aim to understand what elements of a population’s network structure affect its expected socio-diversity *D*_∞_.

Equation (2.3) reveals that the expected socio-diversity is determined by two fundamental factors: first, the per capital innovation rate *α* = *r*|*V*(*G*)|. Unsurprisingly, a higher *per capita* innovation rate leads to greater socio-diversity; second, and most importantly, the network structure *G*, as captured by the structural diversity index Δ(*G*). When Δ(*G*) is high, the expected socio-diversity tends to be high as well, nearing its maximum of 1. Conversely, when Δ(*G*) is low, the expected socio-diversity is low, actually close to the minimum value of 0. This codependence relationship suggests that the structural diversity index Δ(*G*) captures the connection between network structure, upon which it depends, and socio-diversity, which it influences.

**Figure 2.**
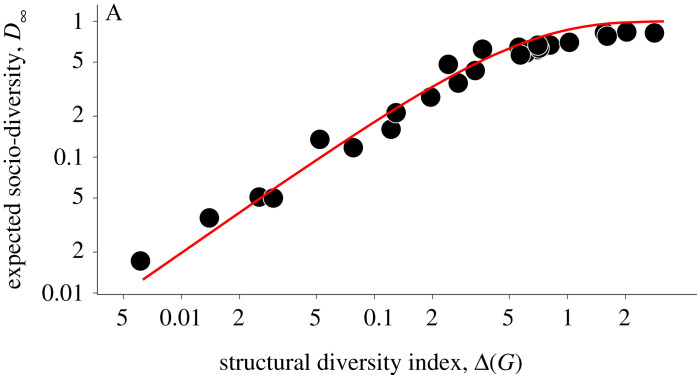
Relationship between expected socio-diversity *D*_∞_ with the structural diversity index Δ(*G*). We simulated the cultural evolution model across various real-world social networks *G* to determine *D*_∞_. Each dot represents a simulation for a distinct social network. The red line depicts the curve 1 − e^−2Δ(*G*)^, our theoretical estimate for *D*_∞_ derived from equation (2.3) using *α* = 1 (or *r* = 1/|*V*(*G*)|). Remarkably, the structural diversity index *predicts* expected socio-diversity levels quite accurately, as evidenced by observations scattering around the red line. See Methods for simulation parameters and descriptions of the social networks.

Figure 2 plots the relationship between expected socio-diversity *D*_∞_ and the structural diversity index Δ(*G*) for various real-world social networks *G*. In a log-log plot, a clear saturating relationship is found, which aligns with our analytical prediction based on equation (2.3) (see red line).

### 2.2. Amplifiers and suppressors of socio-diversity

Equation (2.3) captures the complex interplay between socio-diversity and network structure by expressing the expected socio-diversity *D*_∞_ as a function of a quantity that only depends on network structure, namely, the structural diversity index Δ(*G*). By increasing the structural diversity index Δ(*G*) in equation (2.3), we observe an increase in expected socio-diversity *D*_∞_. Hence, networks with large structural diversity index tend to favour socio-diversity, whereas those with a small one tend to obstruct it.

But what should be considered ‘large’ or ‘small’ structural diversity indices? Large and small are typically defined with respect to a benchmark. The natural benchmark here is the complete network *K*. The complete network reflects the total absence of social structure. There are no communities, no clusters and no differences between individual social positions; the population is structurally homogeneous. Comparing the structural diversity index of an arbitrary network *G* with that of an equally sized complete network *K* informs us about how the network structure affects the index, and, consequently, socio-diversity.

The structural diversity index of the complete network satisfies Δ(*K*) = 1, independent of its size (see Methods for an explanation). If, for a network structure *G*, we have Δ(*G*) < Δ(*K*) = 1, equation (2.3) suggests that the population’s expected socio-diversity is lower than if the population were unstructured. In other words, all else being equal, the variety of memes in a population with structure *G* is expected to be lower than in a population with no structure. Hence, networks *G* with Δ(*G*) < 1 can be said to (structurally) *suppress socio-diversity* (see [3]). By contrast, networks *G* with Δ(*G*) > Δ(*K*) = 1 can be said to (structurally) *amplify socio-diversity*. Indeed, according to equation (2.3), the population’s expected socio-diversity is higher than if the population were unstructured.

Scale-free networks *G*_*γ*_ are networks characterized by a power-law degree distribution *P*(*k*) ∼ *k*^−*γ*^. In the Methods, we show that the structural diversity index of scale-free networks with exponent *γ*, with 2 ≤ *γ* ≤ 3, satisfies

2.4 $$\Delta (G_{\gamma })\leq |V(G_{\gamma }){|}^{-((3-\gamma )/(\gamma -1))}\leq 1.$$

**Figure 3.**
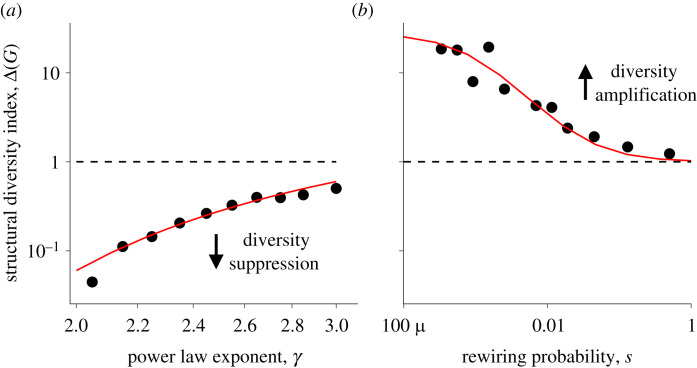
Numerical simulation (dots) and analytical estimates (red lines) of the structural diversity index of (*a*) scale-free and (*b*) Watts–Strogatz networks. These are plotted against (*a*) the power-law exponent *γ* and (*b*) the rewiring probability *s*. In (*a*), the red lines show the equation Δ(*G*_*γ*_) = *b* · |*V*(*G*_*γ*_)|^−*a*·(3−*γ*)/(*γ*−1)^ where *a* and *b* are obtained by ordinary least-squares fit. In (*b*), the red line represents the approximation in equation (2.5). Scale-free networks tend to suppress socio-diversity (Δ(*G*_*γ*_) < 1). Specifically, greater heterogeneity in the degree distribution (i.e. a smaller exponent *γ*) induces greater suppression of socio-diversity (i.e. smaller values of Δ(*G*_*γ*_)). Conversely, Watts–Strogatz networks tend to amplify socio-diversity (Δ(*W*_*s*_) > 1). However, socio-diversity amplification is reduced as more long-range connections are established or/and more randomness is inserted (i.e. as the rewiring probability *s* increases). Electronic supplementary material, movie S1 offers a visual comparison of cultural evolution on scale-free and Watts–Strogatz networks (see Section F.1 of the electronic supplementary material for the movie’s caption). See Methods for simulation parameters.

Therefore, scale-free networks tend to suppress socio-diversity. Moreover, as illustrated in figure 3*a*, diversity suppression intensifies as the scale-free network becomes more degree-heterogeneous (i.e. as the exponent *γ* decreases).

Watts–Strogatz networks *W*_*s*_ interpolate between regular lattices and random networks by means of a parameter *s* ∈ [0, 1], called rewiring probability [23]. The structural diversity index of these networks is roughly

2.5 $$\Delta (W_{s})\approx \frac{s+1/{\left\langle k\right\rangle }^{2}}{s+1/|V(W_{s})|}\geq 1,$$

where 〈*k*〉 denotes the network’s average degree (see Methods). Hence, Watts–Strogatz networks tend to amplify socio-diversity, and this amplification weakens as randomness increases (i.e. as the rewiring probability *s* becomes larger)—see figure 3*b*.

#### 2.2.1. Characteristics of real-world networks that amplify and suppress socio-diversity

**Figure 4.**
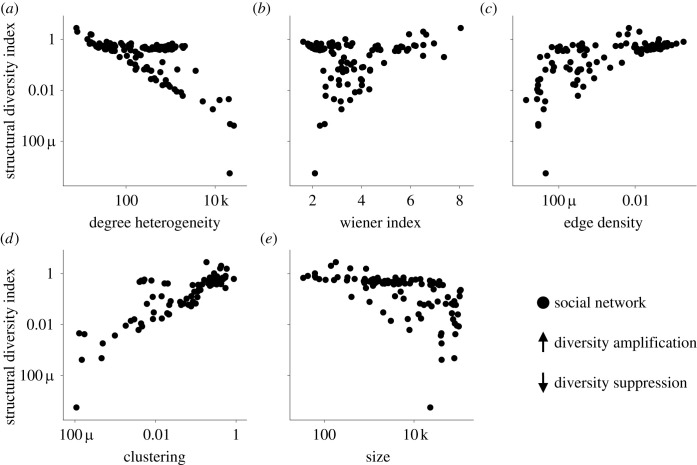
Relationship between the structural diversity index and (*a*) the degree-heterogeneity *κ*(*G*), (*b*) the Wiener index *W*(*G*), (*c*) the edge density *e*(*G*), (*d*) the clustering coefficient *c*(*G*) and (*e*) the size |V(G)| of a network *G* (see main text for definitions). Analysing a variety of social networks we find that high-degree heterogeneity tends to suppress socio-diversity. By contrast, high clustering, Wiener index and edge density tend to amplify it. The effect of network size is more intricate (see text for details). See Methods for descriptions of the networks.

Let us broaden the scope of our analysis from the previous examples and ask: what general characteristics of networks amplify or suppress expected socio-diversity? Figure 4*a*–*e* plots the structural diversity index Δ(*G*) against five well-known properties of social networks—degree-heterogeneity, Wiener index, edge density, clustering and size—for a wide range of real-world social networks *G*. Table 1 presents the outcomes of five regression models. These models help quantify the correlations shown in figure 4 and evaluate their robustness.

**Table 1.** Dependent variable: Log(structural diversity index). The regression models elucidate the correlations between the structural diversity index and network characteristics as illustrated in figure 4: (i) the correlations with degree-heterogeneity, the Wiener index, and edge density are robust. (ii) The correlation with clustering fades when accounting for degree-heterogeneity, Wiener index, size and edge density. (iii) The correlation with size reverses when factoring in other network characteristics. All variables are standardized for a direct comparison between regression coefficients. See main text for interpretations of these results and Methods for technical details about the regressions.

variable	model 1	model 2	model 3	model 4	model 5
log(degree heterogeneity)	−0.67***				−0.56***
	(0.07)				(0.06)
log(wiener index)		0.03			0.37***
		(0.09)			(0.08)
log(edge density)			0.65***		1.38***
			(0.07)		(0.12)
log(clustering)				0.84***	0.02
				(0.05)	(0.06)
log(size)					0.70***
					(0.07)
no. observations	119	119	119	119	119
*R*^2^	0.45	0.0	0.43	0.72	0.93
adjusted *R*^2^	0.44	0.0	0.42	0.71	0.92

***p < 0.001, **p < 0.01, *p < 0.1.

Figure 4*a* reveals a strong negative correlation between the structural diversity index Δ(*G*) and degree-heterogeneity *κ*(*G*), measured as the ratio of the second and first moments of *G*’s degree distribution [52]. As degree heterogeneity increases, the structural diversity index decreases. This correlation is quite robust. It persists even after accounting for other network properties (see table 1) and evaluating alternative measures of inequality in the distribution of connection, such as the Gini Index (see Section B of the electronic supplementary material). At its core, this suggests that high-degree heterogeneity tends to suppress socio-diversity. This phenomenon has an intuitive explanation: large-degree vertices (‘VIPs’, ‘hubs’, ‘influencers’ or ‘hyperinfluentials’ [53]) are crucial—either as initiators or early adopters—in triggering large imitation cascades [53]. This eventually ends up reducing socio-diversity.

Figure 4*b* portrays a positive correlation between the structural diversity index Δ(*G*) and the Wiener index *W*(*G*). Specifically, when the Wiener index has high values, the structural diversity index also tends to be high. The regressions in table 1 confirm this observation. Moreover, Model 5 in this same table reveals an increase of this correlation when controlling for the effects of other network attributes. From a wider viewpoint, these findings indicate that large network distances between individuals tend to amplify socio-diversity. The reason is intuitive: large distances obstruct meme spreading, a phenomeon testified by the geographical clustering of most cultural forms.

Figure 4*c* and Model 3 in table 1 reveal a positive relationship between the structural diversity index and edge density *e*(*G*), defined as the proportion of existing edges to potential edges within the network. Table 1 (Model 5) demonstrates that this correlation intensifies when accounting for other network characteristics, suggesting that edge density may play an important role in the regulation of socio-diversity. Broadly, in a similar vein to how it fosters meritocracy [54], edge density appears to encourage socio-diversity. These findings align with theories arguing that a greater number of connections reduces the influence of each individual connection, thus diminishing the likelihood of large imitation cascades [12].

Figure 4*d* shows a positive correlation between the structural diversity index Δ(*G*) and clustering, as measured by the clustering coefficient *c*(*G*) [23]. On the surface, higher levels of clustering imply a larger structural diversity index. However, Model 5 of table 1 highlights that this correlation fades when factoring in other network characteristics. This suggests that these network characteristics might mediate the amplifying effect of clustering on socio-diversity. To break it down, a large network with high average inter-node distances and significant edge density can support socio-diversity irrespective of its clustering levels. However, in our dataset, most dense networks exhibit high clustering. Consequently, it is complicated to disregard the significance of clustering entirely. This viewpoint is further supported by a straightforward mechanism connecting clustering with socio-diversity: clusters, when they are meme-homogeneous, obstruct consensus formation by increasing the persistence of individual memes and decreasing the exposure to new memes.

Finally, figure 4*e* highlights a negative correlation between the structural diversity index and network size, suggesting that large networks might suppress socio-diversity. However, a closer look (see table 1, Model 5), reveals a shift to a positive correlation when factoring in all the discussed network characteristics. This sheds some light on the nuanced relationship between the structural diversity index and network size: Size intrinsically boosts the index, perhaps due to factors like increased overall innovation in larger networks. Yet, as networks grow, they become more sparse because maintaining connections is costly. And this decrease in edge density is likely responsible for the initial negative correlation observed in figure 4*e*.

## 3. Discussion

Understanding the interplay between network structure and socio-diversity is crucial, as the latter has numerous positive and negative implications for society. In this article, we have made some steps to understand this. We have found that: (i) A simple index, the structural diversity index, captures the complex interplay between network structure and socio-diversity. (ii) Network characteristics can amplify or suppress socio-diversity: high-degree heterogeneity, as in scale-free networks, tends to suppress it, while high local clustering, large inter-node distances, and significant edge density tend to amplify it. For clarity, we explored the voter model, one of the simplest models of cultural evolution. However, in Section C of the electronic supplementary material, we show that qualitatively similar results hold for other fundamental models such as Axelrod’s model [25], Sznajd’s model [55] and the (discrete) bounded confidence model [56,57] (see also the review in [58]).

**Figure 5.**
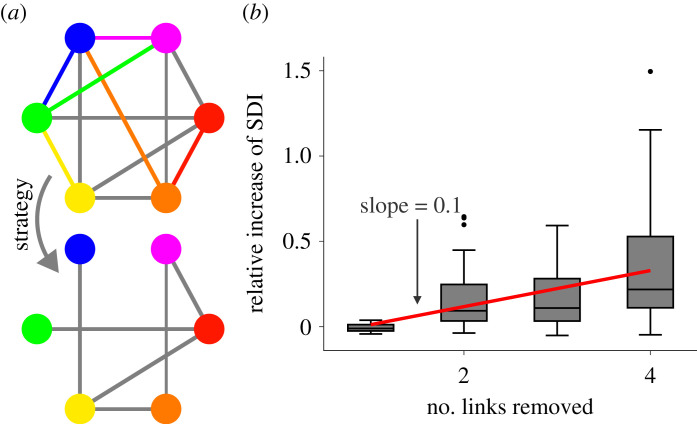
Removing links to highly connected individuals may increase the structural diversity index. (*a*) Illustration of a simple strategy to raise the structural diversity index: each individual in the network *G* (top) removes links to her *h* = 1 most connected neighbours (tie sorting is random), resulting in the network *G*_*h*_ (bottom). (*b*) Percentage change (Δ(*G*_*h*_) − Δ(*G*))/Δ(*G*) in the structural diversity index after applying the procedure outlined in (*a*) to a network *G*. This simple procedure leads to remarkable increases of the structural diversity index, even when the number of removed connections per individual is small. On average, increasing *h* by one leads to a 10% increase in the index. See Methods for details about network data, box plots and regression values.

Our work suggests numerous future research directions (see also Section E of the electronic supplementary material). First, an understanding of the consequences that specific characteristics of networks have for socio-diversity implies opportunities for change. For example, our results hint towards possible ways of transforming social networks to sustain greater socio-diversity. For instance, when an increase in degree-heterogeneity causes a reduction in socio-diversity, a simple, decentralized strategy such as ‘stop following the *h* most connected VIPs in your social network channel’ (or, for short, ‘don’t follow leaders’ [59]) can be surprisingly effective in sustaining it, as shown in figure 5. Future research may explore further kinds of network modification strategies that amplify or suppress socio-diversity. For example, how can one leverage the fact that clustering amplifies socio-diversity?

Second, and most importantly, our findings are primarily rooted in models of cultural evolution, rather than in actual experimental data. It is, therefore, essential to remember that all models inherently simplify the complexities of human interactions. As such, empirical data may reveal nuances that go beyond the narratives presented in our study. Thus, we encourage follow-up research to validate our conclusions through lab or online experiments. In essence, a pivotal question lingers: Can the insights about the relationship between network structure and socio-diversity gained from our modelling and simulations be replicated in an experimental environment?

A third key direction of future research is to deepen our understanding of what levels of socio-diversity are beneficial for distinct social systems. Our work illustrates how one can change social networks to promote or reduce socio-diversity, but it does not tackle whether more or less diversity is desirable. Socio-diversity can bring both advantages and challenges: while an overabundance might lead to division and conflict, too little could hinder innovation and collective intelligence. Pinpointing the ideal balance is complex, necessitating a careful consideration of socio-diversity’s multifaceted effects. Nevertheless, given its profound implications for societal dynamics, it is high time that we appreciate the importance of socio-diversity and explore new ways of shaping it.

## 4. Methods

### 4.1. Proof of equation (2.3)

We will present a direct proof of this equation (2.3), based on the fundamentals of random walk theory.

The proof interlinks the model of cultural evolution discussed above with the concept of an *r*-random walk on a graph *G*. This *r*-random walk closely resembles a conventional random walk, but with an added twist: at every step, there is a probability *r* of the walk ‘halting’. For a more vivid picture, imagine, as Karl Pearson and Lord Rayleigh [19,60], a drunkard wandering through an urban street network. At every intersection, he randomly selects a street and heads towards the next crossing. The nuance in the *r*-random walk lies here: on any given street, the drunkard might come across a bar he fancies with probability *r*, leading him to leave the street network indefinitely.

The core relationship between *r*-random walks and the cultural evolution model discussed above is summarized in the following equation. This equation is based on the principle of ‘voter model duality’ [49]. It is a straightforward generalization of Aldous’ findings for traditional random walks [61]:

4.1 $$1-D(t)=p_{r}(t).$$

In this context, 1 − *D*(*t*) represents the likelihood that, at time step *t* in the cultural evolution model, a pair of randomly chosen individuals both exhibit the same meme. On the other hand, *p*_*r*_(*t*) indicates the probability that two *r*-random walks, started with uniform probability across the vertices of *G*, meet before completing *t* steps.

Equation (4.1) leaves us with the task of understanding *p*_*r*_(*t*). Since it simplifies the argument and since our main interest concerns the large-time behaviour of the system, we focus on $p_{r}(\infty )=\lim_{t\to \infty }p_{r}(t)$. This effectively means that we are exploring the probability that two *r*-random walks on the graph *G* meet before either one halts.

In mathematical symbols, ‘the probability that two *r*-random walks on the graph *G* meet before either one halts’ can be expressed as:

4.2 $$p_{r}(\infty )=P(M_{G}<\min(S_{1},S_{2})).$$

In this equation, *M*_*G*_ is the meeting time of the graph *G*. This quantity has been defined as the number of steps before two uniformly started *traditional* random walks on *G* visit the same vertex simultaneously. Meanwhile, *S*_1_ and *S*_2_ are geometric random variables with a success probability *r*. These random variables count the number of steps taken by the first and second random walks, respectively, before they come to a halt.

To derive equation (2.3), we approximate

$$P(M_{G}<\min(S_{1},S_{2}))\approx P\left(\langle M_{G}\rangle <\min(S_{1},S_{2})\right).$$

Section A of the electronic supplementary material discusses this approximation in detail. Next, since *S*_1_ and *S*_2_ are geometrically distributed random variables with success probability *r*, $\min(S_{1},S_{2})$ is a geometrically distributed random variable with success probability *q*(*r*) = 2*r* − *r*^2^. Therefore,

$$P\left(\langle M_{G}\rangle <\min(S_{1},S_{2})\right)={\left(1-q(r)\right)}^{\left\langle M_{G}\right\rangle }\approx e^{-q(r)\langle M_{G}\rangle }.$$

When *r* ≪ 1, *q*(*r*) ≈ 2*r*. Hence, $e^{-q(r)\langle M_{G}\rangle }\approx e^{-2r\langle M_{G}\rangle }$. The hypothesis *r* ≪ 1 is convenient for presentation because it makes interpretation more straightforward. However, it is not necessary for the paper’s conclusions to hold. In fact, replacing 2*r* by *q*(*r*) = 2*r* − *r*^2^ yields very similar results.

Drawing upon our prior arguments, we have established

4.3 $$p_{r}(\infty )=P(M_{G}<\min(S_{1},S_{2}))\approx e^{-2r\langle M_{G}\rangle }.$$

Now, equation (4.1) shows that 1 − *D*(*t*) = *p*_*r*_(*t*). Therefore,

4.4 $$p_{r}(\infty )=\lim_{t\to \infty }p_{r}(t)=\lim_{t\to \infty }1-D(t)=1-\lim_{t\to \infty }\sum_{s\leq t}D(s)=1-D_{\infty }.$$

From this relationship and equation (4.3), it is clear that

4.5 $$D_{\infty }=1-p_{r}(\infty )=1-e^{-2r\langle M_{G}\rangle }.$$

Finally, replacing the innovation rate *r* with the *per capita* innovation rate *α* = *r*|*V*(*G*)| and recalling that the structural diversity index is defined as the ratio Δ(*G*) = 〈*M*_*G*_〉/|*V*(*G*)|, we obtain the desired equation

4.6 $$D_{\infty }=1-e^{-2\alpha \Delta (G)}.$$

### 4.2. Structural diversity index of complete, scale-free and Watts–Strogatz networks

For a network *G*, we defined the structural diversity index by $\Delta (G)=\langle M_{G}\rangle /|V(G)|$, where *M*_*G*_ is the meeting time of two (uniformly started) random walks on *G* and |V(G)| is *G*’s vertex count. Let us discuss estimates for the structural diversity index of complete, scale-free, and Watts–Strogatz networks.

#### 4.2.1. Complete networks

For the complete network *K* with vertices, the structural diversity index can be computed exactly. In each step, two random walks on *K* have a probability 1/|V(K)| of moving to the same vertex and, hence, of meeting. Consequently, the meeting time *M*_*K*_ of the walks is geometrically distributed with success probability 1/|V(K)|. In particular, $\left\langle M_{K}\rangle =|V(K)\right|$.

#### 4.2.2. General bounds

For a general network *G*, exact analytical expressions for the meeting time *M*_*G*_ are not in reach. However, good upper and lower bounds are available.

On the one hand, Cooper *et al.* [62] provide an upper bound for the average meeting time 〈*M*_*G*_〉

4.7 $$\langle M_{G}\rangle =O\left(\frac{1}{1-\lambda_{2}}\left(2\log(|V(G)|)+|V(G)|\frac{{\left\langle k\right\rangle }^{2}}{\left\langle k^{2}\right\rangle }\right)\right).$$

Herein, *O* is the standard asymptotic notation for ‘is asymptotically dominated by’; 〈*k*〉 and 〈*k*^2^〉 denote, respectively, the first and second moments of *G*’s degree distribution; and *λ*_2_ indicates the second largest eigenvalue of the transition matrix *P* of the random walk on *G* (i.e. the matrix P(i,j)=1/deg⁡(i) for all vertices *i*, *j* of *G*).

On the other hand, Aldous [47] provides a lower bound for the average meeting time 〈*M*_*G*_〉

4.8 $$\langle M_{G}\rangle =\Omega \left(\frac{\left|E(G)\right|}{D_{\max}}\right)$$

Herein, *Ω* is the standard asymptotic notation for ‘asymptotically dominates’; $D_{\max}$ is the maximum degree in the graph *G*; and |E(G)| is the number of edges.

#### 4.2.3. Scale-free networks

For a scale-free network *G*_*γ*_ with exponent *γ*, different bounds for 1/(1 − *λ*_2_) exist, depending on the model with which the network is constructed [63,64]. However, such bounds are typically polynomials in $\log(|V(G_{\gamma })|)$. Therefore, neglecting logarithmic terms, equation (4.7) suggests that $\left\langle M_{G_{\gamma }}\rangle =O(|V(G_{\gamma }|){\left\langle k\right\rangle }^{2}/\langle k^{2}\rangle \right)$. Hence, for large networks *G*_*γ*_, we have

4.9 $$\Delta (G_{\gamma })\leq \frac{\left\langle k^{2}\right\rangle }{{\left\langle k\right\rangle }^{2}}.$$

Herein, 〈*k*〉 and 〈*k*^2^〉 denote the first and second moments of *G*_*γ*_’s degree distribution. Note that 〈*k*〉 and 〈*k*^2^〉 depend on the exponent *γ*. We have calculated their values in terms of $\left|V(G_{\gamma })\right|$ and *γ* following the guidelines provided in ch. 6.4 of [52]. This yields the bound for Δ(*G*_*γ*_) reported in equation (2.4)

4.10 $$\Delta (G_{\gamma })\leq \left\{ \begin{array}{cc} 1\; & \text{if }\gamma \geq 3\;\\ |V(G_{\gamma }){|}^{-((3-\gamma )/(\gamma -1))}\; & \text{if }2<\gamma <3\;\\ |V(G_{\gamma }){|}^{-1}\; & \text{if }\gamma \leq 2\; \end{array}\right..$$

#### 4.2.4. Watts–Strogatz networks

A Watts–Strogatz network *W*_*s*_ with rewiring probability *s* and average degree 〈*k*〉 has $\left|E(W_{s})|=\langle k\rangle |V(W_{s})\right|$ edges and maximum degree $D_{\max}\approx \langle k\rangle$. Therefore, according to equation (4.8), the average meeting time satisfies $\left\langle M_{W_{s}}\rangle =\Omega (|V(W_{s})|\right)$. Consequently, for large networks *W*_*s*_, we retrieve the lower bound reported in equation (2.5)

The following explicit formula captures the relationship between the structural diversity index and the rewiring probability *s* fairly well

4.12 $$\Delta (W_{s})\approx \frac{s+1/{\left\langle k\right\rangle }^{2}}{s+1/|V(W_{s})|}.$$

However, we could not find a theoretical derivation of this approximate relationship.

### 4.3. Network data

All networks have been handled using graph-tool [24] or networkx [65]. The network data employed in this work are described in Section B of the electronic supplementary material, and are freely available at https://networks.skewed.de. A Python package enabling the fast numerical computation of the structural diversity index developed by the first author is available through PyPi https://pypi.org/project/structural-diversity-index/. See Section D of the electronic supplementary material for more information.

### 4.4. Parameters and specifications for figures

#### 4.4.1. Figure 2

For selected real-world networks *G* from our network dataset (see table B.1 and table B.2 of the electronic supplementary material for details), we computed the expected socio-diversity *D*_∞_ by simulating the cultural evolution model with parameter r=1/|V(G)| for 10⋅|V(G)| steps 20 times; (ii) the structural diversity index Δ(*G*) by simulating 10^4^ realizations of *M*_*G*_ and taking the average. Error bars are smaller than the sizes of symbols.

#### 4.4.2. Figure 3

Simulations were performed on scale-free networks with *N* = 10^3^ vertices and minimum degree *m* = 4 and on Watts–Strogatz networks with *N* = 10^3^ vertices and average degree 〈*k*〉 = 6. We computed the structural diversity index Δ(*G*) by simulating 10^4^ realizations of *M*_*G*_ and averaging. Error bars are smaller than the size of the symbols. The parameters *a* and *b* in (A) were obtained by an ordinary least-squares fit of $\log(\Delta (G_{\gamma }))$ against $\log(|V(G_{\gamma }){|}^{-(3-\gamma )/(\gamma -1)})$. The fit yields *a* = 0.3 and *b* = 6.2 with *R*^2^ = 0.91.

#### 4.4.3. Figure 4

For each real-world network *G* in our network dataset (see Table B.1 and Table B.2 of the electronic supplementary material for details), we calculated (i) the structural diversity index, (ii) degree-heterogeneity, (iii) Wiener index, (iv) edge density, (v) clustering and (vi) network size. We provide definitions of these quantities in the text and in Section B of the electronic supplementary material. We computed the structural diversity index and Wiener index in the same way as outlined for figure 2; clustering using algorithms from graph-tool [24]; degree-heterogeneity, edge density and size by evaluating simple mathematical expressions.

#### 4.4.4. Figure 5

For selected real-world networks *G* in our network dataset (see Table B.1 and Table B.2 of the electronic supplementary material for details) and each *h* = 1, 2, 3, 4, we obtained the network *G*_*h*_ through the edge removal procedure described in figure 5*a*. Specifically, *G*_*h*_ is the largest connected component of the network obtained by the procedure in figure 5*a*. For each network *G*_*h*_, we computed the structural diversity index by simulating 10^4^ realizations of $M_{G_{h}}$ and averaging them. Before creating the plot in figure 5*b*, we cleaned the data by discarding some ‘pathological cases’. First, we discarded all networks with |*V*(*G*_*h*_)| < |*V*(*G*)|/4. These networks were too affected by edge removal for comparisons to be meaningful. Second, we filtered out outliers, i.e. networks such that the relative variation of the structural diversity index (Δ(*G*_*h*_) − Δ(*G*))/Δ(*G*) deviated more than 1 s.d. from the average relative variation of the sample. This procedure discards just a few (about 4) networks for each value of *h*. The discarded networks all follow a common pattern: the relative variations in their structural diversity indices are anomalously large because of specific structural features. Such anomalous cases are not interesting for our statistical study. This is why we discarded them. The red line is fitted using the ordinary least-squares method (*a* = 0.105, *R*^2^ = 0.223).

#### 4.4.5. Table 1

The coefficients, standard errors and *p*-values displayed in table 1 are obtained by running ordinary least-square regressions on log-transformed standardized data. Due to space limitations, we excluded the regression analysis of structural diversity against size, as it was considered the least relevant. The network sample used in the regression is the same as that used in figure 4.

#### 4.4.6. Computing realizations of *M*_*G*_

A realization of *M*_*G*_ is computed by simulating two random walks on the graph *G*. The simulation is run for $s_{\max}=100\cdot |V(G)|$ steps. If the random walks do not meet within *s*_max_ steps, we estimate the value of *M*_*G*_. For this, we use the fact that *M*_*G*_ is approximately geometrically distributed (see Section A of the electronic supplementary material). Specifically, we estimate the geometric distribution that best approximates *M*_*G*_. Then, we sample from this geometric distribution conditioned on the fact that the sampled value should exceed *s*_max_.

## Data Availability

A Python package enabling the fast numerical computation of the structural diversity index developed by the first author is available through PyPi https://pypi.org/project/structural-diversity-index/. A detailed tutorial on how to use the scripts is available on the *eth-coss* public GitHub repository (see https://github.com/ethz-coss/Structural-diversity-index) and the full documentation of the scripts can be found on ReadTheDocs (see https://rse-distance.readthedocs.io/en/latest/). The network data employed in this study are freely available at https://networks.skewed.de. Supplementary material is available online [66].

## Footnotes

1 The memes considered in this paper should not be confused with the homonymous ‘memes’ circulating on social media. Social media ‘memes’ are funny internet content, while this paper’s ‘memes’ are the building blocks of culture.

2 This is not surprising: ‘Understanding the factors determining biodiversity is arguably the most fundamental problem in ecology and conservation biology’ [4], in contrast to the less central role of socio-diversity studies in many social sciences.

3 Innovation need not imply a groundbreaking cultural shift but might just be a nuanced variation of an existing meme.

## References

[1] Dawkins R. 1990 The Selfish Gene, 2nd edn. Oxford, UK: Oxford University Press.

[2] Nowak MA. 2006 Evolutionary dynamics: exploring the equations of life. Cambridge, UK: Belknap Press.

[3] Lieberman E, Hauert C, Nowak MA. 2005 Evolutionary dynamics on graphs. Nature **433**, 312-316. (10.1038/nature03204) 15662424

[4] Bastolla U, Fortuna MA, Pascual-García A, Ferrera A, Luque B, Bascompte J. 2009 The architecture of mutualistic networks minimizes competition and increases biodiversity. Nature **458**, 1018-1020. (10.1038/nature07950) 19396144

[5] Boyd R, Richerson PJ. 1988 Culture and the evolutionary process. Chicago, IL: University of Chicago Press.

[6] Simmel G. 1957 Fashion. Am. J. Sociol. **62**, 541-558. (10.1086/222102)

[7] Granovetter M. 1985 Economic action and social structure: the problem of embeddedness. Am. J. Sociol. **91**, 481-510. (10.1086/228311)

[8] Christakis NA, Fowler JH. 2007 The spread of obesity in a large social network over 32 years. N. Engl. J. Med. **357**, 370-379. (10.1056/NEJMsa066082) 17652652

[9] Christakis NA, Fowler JH. 2008 The collective dynamics of smoking in a large social network. N. Engl. J. Med. **358**, 2249-2258. (10.1056/NEJMsa0706154) 18499567 PMC2822344

[10] Hauert C, Doebeli M. 2004 Spatial structure often inhibits the evolution of cooperation in the snowdrift game. Nature **428**, 643-646. (10.1038/nature02360) 15074318

[11] Nowak MA. 2006 Five rules for the evolution of cooperation. Science **314**, 1560-1563. (10.1126/science.1133755) 17158317 PMC3279745

[12] Granovetter M. 1978 Threshold models of collective behavior. AJS **83**, 1420-1443. (10.1086/226707)

[13] Centola D. 2010 The spread of behavior in an online social network experiment. Science **329**, 1194-1197. (10.1126/science.1185231) 20813952

[14] Ugander J, Backstrom L, Marlow C, Kleinberg J. 2012 Structural diversity in social contagion. Proc. Natl Acad. Sci. USA **109**, 5962-5966. (10.1073/pnas.1116502109) 22474360 PMC3341012

[15] Onnela JP, Saramäki J, Hyvönen J, Szabó G, Lazer D, Kaski K, Kertész J, Barabási AL. 2007 Structure and tie strengths in mobile communication networks. Proc. Natl Acad. Sci. USA **104**, 7332-7336. (10.1073/pnas.0610245104) 17456605 PMC1863470

[16] Granovetter MS. 1973 The Strength of Weak Ties. AJS **78**, 1360-1380. (10.1086/225469)

[17] Lazer D, Friedman A. 2007 The network structure of exploration and exploitation. Admin. Sci. Quart. **52**, 667-694. (10.2189/asqu.52.4.667)

[18] Mason W, Watts DJ. 2012 Collaborative learning in networks. Proc. Natl Acad. Sci. USA **109**, 764-769. (10.1073/pnas.1110069108) 22184216 PMC3271930

[19] Rayleigh. 1905 The problem of the random walk. Nature **72**, 318-318. (10.1038/072318a0)

[20] Levin DA, Peres Y. 2017 Markov chains and mixing times. Boston, MA: American Mathematical Society.

[21] Appadurai A. 1996 Modernity At Large: Cultural Dimensions of Globalization. Minneapolis: University of Minnesota Press.

[22] Barabási AL, Albert R. 1999 Emergence of scaling in random networks. Science **286**, 509-512. (10.1126/science.286.5439.509) 10521342

[23] Watts DJ, Strogatz SH. 1998 Collective dynamics of ‘Small-World’ networks. Nature **393**, 440-442. (10.1038/30918) 9623998

[24] Peixoto T. 2014 The Graph-Tool Python library. (10.6084/m9.figshare.1164194.v14).

[25] Axelrod R. 1997 The dissemination of culture: a model with local convergence and global polarization. J. Conflict Resol. **41**, 203-226. (10.1177/0022002797041002001)

[26] Weitzman ML. 1992 On diversity*. Quart. J. Econ. **107**, 363-405. (10.2307/2118476)

[27] Huckfeldt RR, Johnson PE, Sprague JD. 2004 Political disagreement: the survival of diverse opinions within communication networks. Cambridge, UK: Cambridge University Press.

[28] Stirling A. 2007 A general framework for analysing diversity in science, technology and society. J. R. Soc. Interface **4**, 707-719. (10.1098/rsif.2007.0213) 17327202 PMC2373389

[29] Klemm K, Eguíluz VM, Toral R, Miguel MS. 2003 Global culture: a noise-induced transition in finite systems. Phys. Rev. E **67**, 045101. (10.1103/PhysRevE.67.045101) 12786417

[30] Page SE. 2008 The difference. Princeton, NJ: Princeton University Press. Sun, 08/31/2008 - 12:00.

[31] Feldman MP, Audretsch DB. 1999 Innovation in cities:: science-based diversity, specialization and localized competition. Eur. Econ. Rev. **43**, 409-429. (10.1016/S0014-2921(98)00047-6)

[32] Santos FC, Santos MD, Pacheco JM. 2008 Social diversity promotes the emergence of cooperation in public goods games. Nature **454**, 213-216. (10.1038/nature06940) 18615084

[33] Vasconcelos VV, Constantino SM, Dannenberg A, Lumkowsky M, Weber E, Levin S. 2021 Segregation and clustering of preferences erode socially beneficial coordination. Proc. Natl Acad. Sci. USA **118**, e2102153118. (10.1073/pnas.2102153118) 34876514 PMC8685719

[34] Bettencourt LMA, Samaniego H, Youn H. 2014 Professional diversity and the productivity of cities. Sci. Rep. **4**, 5393. (10.1038/srep05393) 24953448 PMC4066264

[35] Gomez-Lievano A, Patterson-Lomba O, Hausmann R. 2016 Explaining the prevalence, scaling and variance of urban phenomena. Nat. Hum. Behav. **1**, 1-6. (10.1038/s41562-016-0012)

[36] Centola D. 2022 The network science of collective intelligence. Trends Cogn. Sci. **26**, 923-941. (10.1016/j.tics.2022.08.009) 36180361

[37] Hong L, Page SE. 2004 Groups of diverse problem solvers can outperform groups of high-ability problem solvers. Proc. Natl Acad. Sci. USA **101**, 16 385-16 389. (10.1073/pnas.0403723101) 15534225 PMC528939

[38] Lorenz J, Rauhut H, Schweitzer F, Helbing D. 2011 How social influence can undermine the wisdom of crowd effect. Proc. Natl Acad. Sci. USA **108**, 9020-9025. (10.1073/pnas.1008636108) 21576485 PMC3107299

[39] Bernstein E, Shore J, Lazer D. 2018 How intermittent breaks in interaction improve collective intelligence. Proc. Natl Acad. Sci. USA **115**, 8734-8739. (10.1073/pnas.1802407115) 30104371 PMC6126746

[40] Woolley AW, Chabris CF, Pentland A, Hashmi N, Malone TW. 2010 Evidence for a collective intelligence factor in the performance of human groups. Science **330**, 686-688. (10.1126/science.1193147) 20929725

[41] Guzzo RA, Dickson MW. 1996 Teams in organizations: recent research on performance and effectiveness. Annu. Rev. Psychol. **47**, 307-338. (10.1146/annurev.psych.47.1.307) 15012484

[42] Webber SS, Donahue LM. 2001 Impact of highly and less job-related diversity on work group cohesion and performance: a meta-analysis. J. Manage. **27**, 141-162. (10.1177/014920630102700202)

[43] Zenger TR, Lawrence BS. 1989 Organizational demography: the differential effects of age and tenure distributions on technical communication. Acad. Manage. J. **32**, 353-376. (10.2307/256366)

[44] Glaeser EL, Laibson DI, Scheinkman JA, Soutter CL. 2000 Measuring trust*. Quart. J. Econ. **115**, 811-846. (10.1162/003355300554926)

[45] Putnam RD. 2007 E Pluribus Unum: diversity and community in the Twenty-first Century The 2006 Johan Skytte Prize Lecture. Scand. Pol. Stud. **30**, 137-174. (10.1111/j.1467-9477.2007.00176.x)

[46] Milgram S. 1967 The small world problem. Psychol. Today **2**, 60-67. (10.2189/asqu.52.4.667)

[47] Aldous DJ. 1991 Meeting times for independent Markov Chains. Stoch. Process. Appl. **38**, 185-193. (10.1016/0304-4149(91)90090-Y)

[48] Kanade V, Mallmann-Trenn F, Sauerwald T. 2023 On coalescence time in graphs: when is coalescing as fast as meeting? ACM Trans. Algor. **19**, 18:1-18:46. (10.1145/3576900)

[49] Liggett TM. 1985 The voter model. In *Interacting Particle Systems* (ed. TM Liggett), Grundlehren Der Mathematischen Wissenschaften, pp. 226–263. New York, NY: Springer.

[50] Simpson EH. 1949 Measurement of diversity. Nature **163**, 688-688. (10.1038/163688a0)

[51] Aldous D. 2013 Probability approximations via the poisson clumping heuristic. Berlin, Germany: Springer Science & Business Media.

[52] Barrat A, Barthélemy M, Vespignani A. 2008 Dynamical processes on complex networks. Cambridge, UK: Cambridge University Press.

[53] Watts DJ, Dodds PS. 2007 Influentials, networks, and public opinion formation. J. Consumer Res. **34**, 441-458. (10.1086/518527)

[54] Borondo J, Borondo F, Rodriguez-Sickert C, Hidalgo CA. 2014 To each according to its degree: the meritocracy and topocracy of embedded markets. Sci. Rep. **4**, 3784. (10.1038/srep03784) 24445533 PMC3896904

[55] Sznajd-Weron K, Sznajd J. 2000 Opinion evolution in closed community. Int. J. Mod. Phys. C **11**, 1157-1165. (10.1142/S0129183100000936)

[56] Hegselmann R, Krause U. 2005 Opinion dynamics driven by various ways of averaging. Comput. Econ. **25**, 381-405. (10.1007/s10614-005-6296-3)

[57] Lorenz J. 2007 Continuous opinion dynamics under bounded confidence: a survey. Int. J. Mod. Phys. C **18**, 1819-1838. (10.1142/S0129183107011789)

[58] Castellano C, Fortunato S, Loreto V. 2009 Statistical physics of social dynamics. Rev. Mod. Phys. **81**, 591-646. (10.1103/RevModPhys.81.591)

[59] Livan G. 2019 Don’t follow the leader: how ranking performance reduces meritocracy. R. Soc. Open Sci. **6**, 191255. (10.1098/rsos.191255) 31827860 PMC6894586

[60] Pearson K. 1905 The problem of the random walk. Nature **72**, 294-294. (10.1038/072294b0)

[61] Aldous D. 2013 Interacting particle systems as stochastic social dynamics. Bernoulli **19**, 1122-1149. (10.3150/12-BEJSP04)

[62] Cooper C, Elsässer R, Ono H, Radzik T. 2013 Coalescing random walks and voting on connected graphs. SIAM J. Discrete Math. **27**, 1748-1758. (10.1137/120900368)

[63] Gkantsidis C, Mihail M, Saberi A. 2003 Conductance and congestion in power law graphs. In *Proc. of the 2003 ACM SIGMETRICS Int. Conf. on Measurement and Modeling of Computer Systems* SIGMETRICS ’03, pp. 148–159. New York, NY: Association for Computing Machinery.

[64] Mihail M, Papadimitriou C, Saberi A. 2003 On certain connectivity properties of the internet topology. In *44th Annual IEEE Symp. on Foundations of Computer Science, 2003. Proc.*, pp. 28–35. (10.1109/SFCS.2003.1238178)

[65] Hagberg A, Swart PJ, Schult DA. 2008 Exploring network structure, dynamics, and function using NetworkX. Technical Report LA-UR-08-05495; LA-UR-08-5495 Los Alamos National Laboratory (LANL), Los Alamos, NM.

[66] Musso A, Helbing D. 2024 How networks shape diversity for better or worse. *Figshare*. (10.6084/m9.figshare.c.7184003) PMC11285507 39076799

